# Decoding the informational properties of the RNA polymerase II Carboxy Terminal Domain

**DOI:** 10.1186/1756-0500-5-241

**Published:** 2012-05-16

**Authors:** Jim Karagiannis

**Affiliations:** 1 Department of Biology, University of Western Ontario, London, ON, Canada

**Keywords:** Transcription, RNA polymerase II, Carboxy terminal domain, Information theory, Phosphorylation, Kinase, Phosphatase, Fission yeast, Budding yeast

## Abstract

**Background:**

The largest sub-unit of RNA polymerase II, Rpb1p, has long been known to be subject to post-translational modifications that influence various aspects of pre-mRNA processing. However, the portion of the Rpb1p molecule subject to these modifications – the carboxy-terminal domain or CTD – remains the subject of much fascination. Intriguingly, the CTD possesses a unique repetitive structure consisting of multiple repeats of the heptapeptide sequence, Y_1_S_2_P_3_T_4_S_5_P_6_S_7_. While these repeats are critical for viability, they are not required for basal transcriptional activity in vitro. This suggests that – even though the CTD is not catalytically essential – it must perform other critical functions in eukaryotes.

**Presentation of the Hypothesis:**

By formally applying the long-standing mathematical principles of information theory, I explore the hypothesis that complex post-translational modifications of the CTD represent a means for the dynamic “programming” of Rpb1p and thus for the discrete modulation of the expression of specific gene subsets in eukaryotes.

**Testing the Hypothesis:**

Empirical means for testing the informational capacity and regulatory potential of the CTD – based on simple genetic analysis in yeast model systems – are put forward and discussed.

**Implications of the Hypothesis:**

These ideas imply that the controlled manipulation of CTD effectors could be used to “program” the CTD and thus to manipulate biological processes in eukaryotes in a definable manner.

## Background

The proper control of gene expression is crucial for a cell to grow, divide, and respond intelligently to environmental and/or developmental cues. Conversely, improper gene expression can lead to a variety of abnormal metabolic, developmental, and/or physiological states [1-7]. Of particular importance to this regulatory control in eukaryotes is the dynamic modulation of the phosphorylation status of the largest sub-unit of RNA polymerase II (Rpb1p) [8-10].

RNA polymerase II has long been known to exist in both hyper- and hypo-phosphorylated forms (RNA pol II_o_ and RNA pol II_a_, respectively). These two forms result from the regulated phosphorylation and de-phosphorylation of a carboxy-terminal extension found in the Rpb1p sub-unit [8-12]. This extension – referred to simply as the carboxy-terminal domain or CTD – is composed of tandem repeats of the heptapeptide sequence, Y_1_S_2_P_3_T_4_S_5_P_6_S_7_. The phosphorylation status of these repeats (the heptapeptides can be phosphorylated on Tyr-1, Ser-2, Thr-4, Ser-5, and/or Ser-7 residues) is controlled by 1) a sub-family of cyclin dependent kinases and 2) a group of small CTD phosphatases [8,10].

While the number of repeats varies from species to species (15 copies in Microsporidia compared to a total of 52 in humans) the presence of this tandem set of heptapeptide repeats is highly conserved in all fungi, plants, and metazoans [10,13,14]. The fact that they are found in virtually all complex, multicellular eukaryotes, but are absent in less complex organisms such as the red algae and plasmodia, has led some to surmise that the CTD is crucial for the development of increased complexity [14].

The importance of the CTD is also borne out by the observation that – while partial truncations of the CTD sequence can be tolerated – deletion of the CTD in its entirety is invariably lethal [9,10]. Most importantly, however, while the CTD is clearly essential for viability, it is not required for basal transcriptional activity in vitro [8,9,11]. This key observation strongly suggests that – even though the CTD is not catalytically required – it must perform other important functions in eukaryotes.

Using a perspective grounded in information theory I explore the hypothesis that complex post-translational modifications of the CTD represent a viable means for the dynamic and modular regulation of discrete genetic regulatory networks within eukaryotic cells. While empirical evidence supporting a role of the CTD in the regulation of specific pathways has been mounting [15-24], a formal examination of the informational potential of the CTD has not been presented. I begin by using two established informational paradigms – the Shannon and Kolmogorov-Chaitin theories – to describe the informational properties of the CTD; empirical means for directly testing the informational capacity and regulatory potential of the CTD are then discussed. Lastly, the implications of these ideas for the controlled experimental manipulation of the CTD and Rpb1p function are examined.

## Presentation of the hypothesis

### From the Shannon Perspective

While the unique structure of the RNA pol II CTD has long been the object of intense interest, a formal, mathematical examination of the CTD with regards to its informational potential has not been presented. To initiate this process I begin by providing a summary of Shannon information theory as it pertains to the CTD of RNA pol II.

A mathematical conceptualization of both information and communication were first presented by Claude Shannon in his classic 1948 paper “A Mathematical Theory of Communication” [25]. In this manuscript Shannon introduces the quantity, *H* (or entropy), as a measure of information, choice and uncertainty. Using the simplifying assumption that each symbol in a string of characters (i.e. a message) has an equal chance of appearing, *H* can be calculated using the formula.

(1) $$H=L\cdot \log_{2}M$$

where *M* is equal to the number of symbols in the alphabet used to write the message, and *L* is equal to the number of characters in the string. For example, the value of *H* for a 10 character binary string is calculated as.

(2) $$\begin{array}{c} H=10\cdot \log_{2}2\\ =10\;bits \end{array}$$

Thus, a receiver awaiting the communication of the string – and having no prior knowledge as to the contents of the string in question – would receive 10 bits of information upon reading the message. Similarly, the value of *H* for a 12 character string of DNA (constructed using the letters A, G, C, or T), is calculated as

(3) $$\begin{array}{c} H=12\cdot \log_{2}4\\ =24\;bits \end{array}$$

The choice of logarithmic base is arbitrary and simply determines the units of measurement (i.e. bits if the base two is chosen, nats if the base *e* is chosen, and digits if the base 10 is chosen).

Using the above paradigm, it is a relatively simple process to apply these concepts to the RNA pol II CTD. Abstractly, at least, one could consider the CTD as a string consisting of *x* repeats of the heptapeptide sequence, Y_1_S_2_P_3_T_4_S_5_P_6_S_7_. The symbol, or letter, that appears at each position in the string will of course depend on the post-translational modifications of the heptad in question. The CTD can be differentially phosphorylated on Ser-2, Ser-5, and/or Ser-7 residues [4,8-10,12,14,26-28]. In addition to these modifications, P_3_ and P_6_ residues may be in either a *cis* or a *trans* configuration (controlled by peptidyl prolyl *cistrans* isomerases). Taking these facts into consideration, it is apparent that one of a total of 32 possible symbols may appear in each heptad repeat. While further post-translational modifications of the CTD are possible (e.g. glycosylation, Y_1_ phosphorylation, T_4_ phosphorylation) these have not been considered for the sake of simplicity.

Proceeding with this train of thought, we can calculate the quantity, *H*, for the human CTD (which consists of 52 heptad repeats) to be

(4) $$\begin{array}{c} H=52\cdot \log_{2}32\\ =260\;bits \end{array}$$

Thus, one can reason that the human CTD has 260 bits of informational potential.

**Figure 1:**
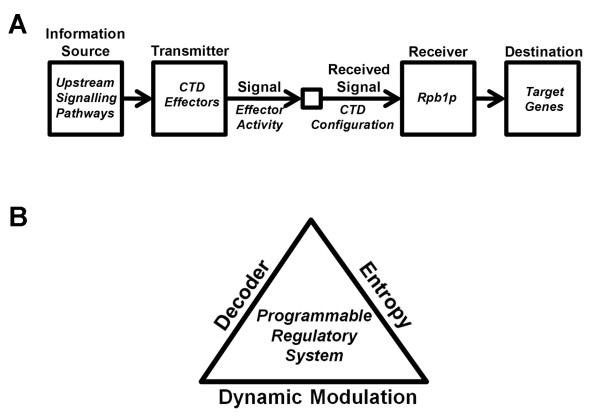
**Harnessing entropy within the CTD to transmit a message. (A)** Dynamic modulation of the CTD seen as a Shannon-type communication system. A noise source is omitted for the sake of simplicity. **(B)** Essential elements of a programmable cellular communication system. The existence of entropy (i.e. “uncertainty”) within a system, together with the ability to 1) modulate the entropy in a controlled manner and 2) decode the signals, would be sufficient to define a “programmable” (by natural selection) regulatory system.

In addition to this potential, it should be noted that all elements of a general communication system as described by Shannon, are present within the eukaryotic cell (Figure 1A). In this biological incarnation of the system, the information source would be comprised of the upstream signalling pathways that converge upon regulatory CTD kinases, phosphatases, and/or *cis*-*trans* isomerases. In this way the message – transmitted through the modulation of the activity of CTD effectors – could be received and decoded by Rpb1p in the form of a discrete CTD phosphorylation pattern. Critically, this decoded message could be used by the cell to influence Rpb1p transcriptional activity in an evolutionarily selectable fashion.

While I have shown that each heptad of the CTD has the potential to encode 5 bits of information, this calculation represents an idealized case where each symbol in the alphabet has an equal chance of appearing at any given position in the string. Since the phosphorylation/*cis-trans* configuration of specific residues in the CTD is not necessarily independent, this idealized scenario is likely not the case in vivo. In cases where each symbol in the alphabet does not have an equal chance of appearing, the quantity *H*, or entropy, can be defined by

(5) $$H=-\sum_{i=1}^{n}p_{i}\cdot \log(p_{i})$$

Where *n* is equal to the number of symbols in the alphabet and *p*_*i*_ represents the probability of the *i*th symbol becoming part of the string. For example, in the binary case, maximum *H* will occur when the two alternate symbols are equally likely to appear. This can be seen from an intuitive standpoint by considering a two symbol system in which one of the symbols never appears. Such a system would be unable to encode information as only strings of a single symbol could be produced. At the other extreme, the same binary system in which both symbols are equally likely, could encode 1 bit/symbol. Intermediate systems (i.e. where one symbol is less likely than the other, but not zero) would be able to encode greater than 0, but less than 1 bit/symbol. Furthermore, in cases where *n* is greater than 2, it can be shown that for any given *n*, *H* will be at a maximum when the probabilities of the symbols (the choice of letters) are equal (i.e. *p*_*i*_ is equal to 1/*n*).

A further concern that must be considered involves the potential for ambiguity in the string. If ambiguity exists, then the amount of information (*R*) transmitted will be determined by the decrease in uncertainty of the receiver according to the equation

(6) $$R=H_{\mathrm{Before}}-H_{\mathrm{After}}$$

For example, if a receiver is expecting a binary string of 20 characters (where *p*_*0*_ = *p*_*1*_ = 0.5), but 3 of the characters are ambiguous upon receipt (i.e. the receiver is unable to determine whether they are 0's or 1's), then the information received is calculated as.

(7) $$\begin{array}{c} R=20\;\log_{2}2-3\;\log_{2}2\\ =17\;bits \end{array}$$

If, on the other hand, the string is sent with no ambiguity then *R* is simply equal to *H*_*Before*_*.*

Given these mathematical constraints – and merging them with our current understanding of the biological reality – it is possible to envision a cellular communication system in which the entropy within the CTD is harnessed to transmit information to the transcriptional machinery. This hypothesis, which I refer to as the “ear of the king” hypothesis (i.e. where the CTD is thought of as a means of gaining access to the “king”, RNA pol II) posits that CTD kinases/phosphatases/*cis*-*trans* isomerases, influenced by upstream signalling pathways, modify the CTD so that the total cellular population of Rpb1p molecules are organized into an ordered, or set of ordered, configurative states (i.e. a discrete set of post-translational modifications specific to a given environmental/developmental/physiological condition). These configurations could in turn modulate the activity of the RNA pol II complex and thus effect discrete changes in the expression of specific gene subsets. In this model each set of ordered configurative states would correspond to a specific expression profile and would provide a selective advantage in a given growth environment.

In effect, this is to say that – depending on the overall activity of the regulatory kinases/phosphatases/*cis*-*trans* isomerases affecting the CTD – RNA pol II could be “programmed” through natural selection to output a specific gene expression profile (Figure 1B). Furthermore, the longer the CTD, the greater the potential entropy and the more diverse the set of configurative states possible. Thus, the correlation between developmental complexity and CTD length are entirely consistent with – and can be logically derived from – these principles.

### From the Kolmogorov-Chaitin Perspective

In addition to the Shannon theory, an independently derived theory of information (referred to as algorithmic information theory, or simply the Kolmogorov-Chaitin theory) has also been presented [26,29-31]. In this theory – just as in the Shannon theory – the uncertainty within a string of characters correlates with the capacity to encode information. In this case the uncertainty is measured by one’s ability to compress or simplify a string.

For example, the string “abcabcabcabcabcabcabcabc” could be compressed to (abc)_8_, whereas a random string (e.g. “arfgjkaaczxfoms”) could not be expressed in any form simpler than merely restating the string. In this paradigm we can informally state that the complexity of a string is equal to the length of the shortest string capable of describing it. Thus, the first example of a string, “abcabcabcabcabcabcabcabc”, would be considered less complex than the second example, “arfgjkaaczxfoms”. Strings are said to be Kolmogorov random if they are incompressible.

Along similar lines to the logic used when discussing the Shannon paradigm, the great potential complexity within the CTD could be harnessed to convey information by “programming” the CTD through the controlled and dynamic modulation of CTD effectors. For example, specific “configurative states” of the CTD (annotated as follows for example; [Y_1:OH_S_2:PO4_P_3:*cis*_T_4_S_5:OH_P_6:*trans*_S_7:OH_]_38_ [Y_1:OH_S_2:OH_P_3:*trans*_T_4_S_5:PO4_P_6:*trans*_S_7:PO4_]_14_) could be seen as selectable “programs” capable of being read by the transcriptional machinery and corresponding to a specific transcriptional output. In this case the quantity of information transmitted could be calculated by determining the difference in complexity between a randomized CTD configuration, and a given non-randomized sequence. This is to say, a change from a randomized CTD configuration to a simpler configuration would constitute a message to Rpb1p to carry out a certain transcriptional program. In this way, controlled modulation of the entropy of the CTD could be used to drive broad developmental/metabolic/physiological changes to gene expression in a selectable manner.

## Testing the hypothesis

### Complexity within the CTD

While the hypothesis presented above might be of some philosophical interest, to be considered a useful theory, it must be used to generate novel and testable predictions. The first prediction that will be addressed relates to the degree of complexity/entropy within the CTD. While high levels of complexity have been shown from a biochemical/molecular perspective (i.e. differential phosphorylation, isomerization, glycosylation) evidence from a more biological or phenotypic perspective is lacking. If the CTD is indeed programmable, and this programming is relevant to phenotypic diversity within an organism, then it should be possible to influence a broad range of distinct phenotypic characteristics by manipulating the configuration of the CTD. For example, differential phosphorylation of the CTD has been shown to be specifically affect the DNA damage response, cell separation, cytokinesis, and meiosis in yeast model systems [16-18,21-24].

A simple scheme to test this prediction in yeast would involve positive selection for CTD configurations able to suppress a panel of conditional mutants. For example, in *Schizosaccharomyces pombe*, a wide array of *ts* mutants affecting processes ranging from cell cycle control, morphogenesis, cytokinesis, DNA repair, to nucleocytoplasmic transport are available. By constructing a library of integrating plasmid vectors – bearing a random set of site-directed CTD mutations capable of replacing the endogenous CTD [21,23] – it would be possible to determine the range of phenotypes suppressible by CTD variants. Precedent for such a scheme has already been established by the observation that *rpb1* mutations (in which Ser-2 residues of the heptad repeats are replaced with alanine) suppress *ts cdc16-116* mutations at the restrictive temperature [21]. If the CTD does indeed possess high degrees of entropy, then it should be possible to obtain distinct configurative states capable of suppressing many of the *ts* mutants in the query panel.

### “Molecular Machine” vs. “Communication Machine”

A second prediction of the hypothesis concerns the responsiveness of the CTD to intra- or extracellular signals. If Rpb1p were simply a “molecular machine“ that used differential phosphorylation to control progress through transcription, then the configuration of the CTD would vary in a manner dependent on the position of Rpb1p in the transcription cycle. Thus, at any instant in time, the complete set of Rpb1p molecules present within a cell would be in any one of a wide variety of potential configurations.

If, on the other hand, the CTD were a “communication machine”, then CTD configuration would vary, at least in part, as a function of environmental condition and/or developmental state. Therefore, one would predict that – as opposed to a haphazard collection of CTD configurations – distinct configurations would emerge as being more highly represented under any given choice of growth parameters. Furthermore, one would expect that any unique set of growth parameters would correlate with a distinct and definable group of over-represented configurations.

While the direct measurement of the complete set of configurative states at any particular instant in time is not feasible with current technologies, indirect evidence could come from systematically assessing phosphorylation patterns using phosphospecific anti-CTD antibodies across a wide variety of growth conditions and in a diverse set of cell types. Some supporting evidence has already appeared in the literature as changes in phosphorylation patterns have been observed in response to DNA damage, heat shock and changes in growth state [15-17].

### Decoding the system

Lastly, if correct in the viewpoint that the Rpb1p CTD is an effective means of cellular communication, then it should be possible to decipher (i.e. decode) the system. By decoding the CTD I do not suggest a detailed – and possibly endless – characterization of the molecular/biochemical details in various cell types and/or environmental conditions. Instead – using yeast model systems – it is suggested that synthetic genetic array (SGA) analysis could be used as an effective tool to decipher the phenotypic effects of specific CTD configurations.

For example, to examine the biological consequences of modulating Ser-2 phosphorylation, site directed mutants at position 2 of the heptad repeats could be used as queries against the array of viable haploid deletion mutants (available in both *Saccharomyces cerevisiae* and *Schizosaccharomyces pombe)*[32]. By examining the phenotype of any individual site-mutant together with its synthetic profile obtained from SGA analysis, and then combining this with expression profiling data, it may be possible to systematically define the relationship between CTD configuration, transcriptional output and phenotypic consequence. Using this knowledge it may be possible to begin deciphering the “programming language” used by a cell to effect broad transcriptional changes.

## Implications of the hypothesis

In the same way that a waterfall possesses potential energy that can be harnessed for useful purposes, it is envisioned that the CTD of RNA pol II possesses an informational potential (i.e. entropy) that – if sufficiently understood – could be harnessed to manipulate biological processes in eukaryotes. For example, if discernable patterns emerge from the experiments described above (i.e. the entropy is decipherable) one could use this information to drive phenotypic alterations. This is to say, one could envision the controlled manipulation of CTD effectors (e.g. through the use of analogue sensitive kinase alleles in unicellular model eukaryotes or drugs in more developmentally complex model eukaryotes) to “program” the CTD and thus to manipulate biological processes in a definable manner. While highly speculative, the feasibility of such an approach is indeed directly testable using technologies available in both budding and fission yeast model systems.

## Abbreviations

RNA: pol II; RNA: polymerase II; CTD: Carboxy terminal domain; SGA: Synthetic genetic array.

## Competing interests

The author declares that he has no competing interests.
